# The use of milrinone in neonates with persistent pulmonary hypertension of the newborn - a randomised controlled trial pilot study (MINT 1): study protocol and review of literature

**DOI:** 10.1186/s40748-018-0093-1

**Published:** 2018-12-03

**Authors:** Afif EL-Khuffash, Patrick J. McNamara, Colm Breatnach, Neidin Bussmann, Aisling Smith, Oliver Feeney, Elizabeth Tully, Joanna Griffin, Willem P. de Boode, Brian Cleary, Orla Franklin, Eugene Dempsey

**Affiliations:** 1 Department of Neonatology, The Rotunda Hospital, Dublin, Ireland; 2 Department of Paediatrics, Royal College of Surgeons, Dublin, Ireland; 3 Division of Neonatology, Stead Family Department of Pediatrics, Iowa City, IA USA; 4 Department of Clinical Research, The Rotunda Hospital, Dublin, Ireland; 5 Department of Neonatology, Radboud University Medical Center, Nijmegen, The Netherlands; 6 Department of Pharmacy, The Rotunda Hospital, Dublin, Ireland; 7 School of Pharmacy, Royal College of Surgeons, Dublin, Ireland; 8 Department of Paediatric Cardiology, Our Lady’s Children’s Hospital Crumlin, Dublin, Ireland; 9 INFANT Centre, University College Cork, Cork, Ireland; 10 Department of Paediatrics and Child Health, University College Cork, Cork, Ireland

**Keywords:** Nitric oxide, Pulmonary hypertension, Milrinone, Phosphodiesterase, Neonates

## Abstract

Persistent pulmonary hypertension of the newborn (PPHN) is a relatively common condition which results in a mortality of up to 33%. Up to 40% of infants treated with nitric oxide (iNO) either have a transient response or fail to demonstrate an improvement in oxygenation. Milrinone, a selective phosphodiesterase 3 (PDE3) inhibitor with inotropic and lusitropic properties may have potential benefit in PPHN. This pilot study was developed to assess the impact of milrinone administration on time spent on iNO in infants with PPHN. This is a multicentre, randomized, double-blind, two arm pilot study, with a balanced (1:1) allocation of 20 infants. In this pilot study, we hypothesise that infants ≥34 weeks gestation and ≥ 2000 g with a clinical and echocardiography diagnosis of PPHN, intravenous milrinone used in conjunction with iNO will result in a reduction in the time spent on iNO. In addition, we hypothesise that milrinone treatment will lead to an improvement in myocardial performance and global hemodynamics when compared to iNO alone. We will also compare the rate of adverse events associated with the milrinone, and the pre-discharge outcomes of both groups. The purpose of this pilot study is to assess the feasibility of performing the trial and to obtain preliminary data to calculate a sample size for a definitive multi-centre trial of milrinone therapy in PPHN.

**Trial registration:** www.isrctn.com; ISRCTN:12949496; EudraCT Number:2014–002988-16.

**Electronic supplementary material:**

The online version of this article (10.1186/s40748-018-0093-1) contains supplementary material, which is available to authorized users.

## Introduction

### Scope of the problem

Persistent pulmonary hypertension of the newborn (PPHN) is a relatively common condition occurring in 0.5 to 7 per 1000 live births and results in a mortality ranging between 4 to 33% [1, 2]. Inhaled nitric oxide (iNO) and extracorporeal membrane oxygenation (ECMO) are the only current therapeutic options that are systematically evaluated in clinical trials [3, 4]. The vasodilatation induced by iNO is mediated by increasing concentrations of the second messengers: cyclic guanyl monophosphate (cGMP) and cyclic adenosine monophosphate (cAMP) in pulmonary vascular smooth muscle. The widespread use of iNO has resulted in a reduction in the need for ECMO; however, mortality and long-term morbidity remain unchanged [1, 4]. In addition, up to 40% of infants treated with iNO either have a transient response or fail to demonstrate an improvement in oxygenation [4, 5]. iNO does not improve myocardial performance in infants with PPHN, which often accompanies the condition and may contribute to mortality. Furthermore, the increasing cost of administering iNO to infants with PPHN may prohibit its use in developing countries. Due to these challenges, there is a real need to evaluate novel approaches to the management of PPHN.

### Pathophysiology and clinical characteristics of PPHN

The condition is clinically characterised by hypoxemic respiratory failure due to the lack of transition of the pulmonary vasculature from a high resistance fetal to a low resistance extra uterine circuit. Pulmonary vascular resistance (PVR) remains high resulting in right to left shunting across the patent foramen ovale (PFO) and the patent ductus arteriosus (PDA) resulting in hypoxemia. On a cellular level, the condition is characterised by marked endothelial dysfunction, with an excess of vasoconstrictor substances (phosphorylated myosin light chains (MLC-P), endothelin 1 (ET-1) and reactive nitrogen species) over vasodilator compounds (cGMP, cAMP, and myosin light chain phosphatase (MLCP)) [6, 7]. Chronic exposure to hypoxia may lead to pulmonary vessel wall thickening with increased connective tissue deposition and neo-muscularisation. This results in a more permanent vascular change termed pulmonary vascular remodelling [8].

### Myocardial dysfunction associated with PPHN

Right (RV) and left (LV) ventricular function may be compromised in PPHN as a result of increased RV afterload and reduced LV preload (due to the reduced pulmonary venous return) [9]. The effects of a pressure-loaded, dilated right heart include a shift in the interventricular septum and compression of the left ventricle, both resulting in decreased LV filling and hence cardiac LV output (LVO). The low cardiac output state resulting from reduced LV preload can lead to a fall in blood pressure in infants with PPHN necessitating the use of vasoactive inotropes such as dopamine and adrenaline. Animal data suggest that these inotropes raise systemic and pulmonary vascular resistance and may further contribute to RV compromise in the setting of PPHN [10, 11]. Several studies have demonstrated the association of a low cardiac output in the setting of PPHN with morbidity and mortality [12–14].

### The potential use of milrinone in PPHN

Cyclic nucleotide phosphodiesterases (PDE) are a family of enzymes that hydrolyse the phosphodiester bond in cAMP and cGMP thereby inhibiting their pulmonary vasodilator properties. Two isoforms, PDE3 and PDE5 are abundantly present in the neonatal lung and play a key role in the pathogenesis of PPHN [15]. PDE3 has a predominant hydrolysing effect on cAMP. Milrinone is a selective PDE3 inhibitor with pharmacological effects including relaxation of vascular smooth muscle, enhanced myocardial contractility (inotropy) and improved myocardial relaxation (lusitropy) [16, 17]. In the newborn lamb, intravenous milrinone augments the action of prostaglandins (PGI_2_) on pulmonary vasculature by significantly shortening the onset and prolonging the duration and degree of pulmonary vasodilation produced by PGI_2_ [18, 19]. Milrinone may also exhibit synergistic effects with iNO in lowering PVR [20, 21].

The use of milrinone is established in neonates and children following cardiac surgery for the prevention of low cardiac output syndrome and the treatment of pulmonary hypertension [22, 23]. Its use in the setting of PPHN of the newborn is limited to case series demonstrating an improvement in oxygenation when used in infants with PPHN failing to respond to iNO [24, 25]. Through its lusitropic and inotropic properties, milrinone may be an effective agent in addressing RV and LV dysfunction in the setting of PPHN.

### Lack of consensus in the management of PPHN

Despite recent advances in the management of PPHN, the risk of mortality and adverse neurological sequelae remains high and often prompt neonatologists to institute additional therapies. However, there is no consensus on the choice of therapeutic interventions in addition to iNO. Characterizing variation in practices is a crucial step toward improved patient outcome. As a result, our group conducted a prospective cross-sectional online survey of neonatologists to evaluate intensive care practices in Canada and the Australia-New Zealand region (AUS-NZ).

A 35-item questionnaire was developed, validated, and piloted to collect information on diagnosis, inhaled nitric oxide (iNO) practices, alternative vasodilators or cardiotropes, and echocardiography. Variation among survey respondents as well as intergroup comparison was performed. Data were collected from 217 respondents. Echocardiography and arterial blood gas were the most common diagnostic tests used to assess the severity of PPHN. iNO administration was more frequently scrutinized in Canada (36% versus 10% [AUS-NZ], *p* < 0.001) due to the higher cost of the therapy in North America. As a consequence, Canadian neonatologists reported higher use of intravenous milrinone (*p* < 0.001), vasopressin (*p* = 0.02), and inhaled prostacyclin (*p* = 0.02), but lower use of sildenafil (*p* = 0.01) for refractory pulmonary hypertension. A greater proportion of neonatologists in AUS-NZ were trained to perform echocardiography (*p* < 0.001) to optimize treatment decisions. As a result of this wide variation in the management of PPHN, there is a need to provide more guidance regarding principles of management while recognizing the dynamic nature of cardiopulmonary physiology in individual patients [26].

### Pharmacokinetics of milrinone in PPHN

The pharmacological profile of milrinone in persistent pulmonary hypertension of the newborn, its short term outcomes and safety profile was recently delineated by our group in an open label prospective pharmacological study of 11 neonates with PPHN [27]. The study included infants ≥34 weeks gestation with birth weights ≥1500 g; less than 10 days old and within 24 h of admission; echocardiography diagnosis of PPHN [right to left shunt at PFO or PDA level and/or severe tricuspid regurgitation]; absence of congenital heart disease and an indwelling arterial line. The most common reasons for PPHN were meconium aspiration syndrome and hypoxic-ischemic encephalopathy. The median ages at administration of iNO and milrinone were 7.5 h (range 4–22) and 14 h (range 10–30); therefore, the interval between these co-treatments was a median of 7 h (4.5–16). All patients required 100% oxygen prior to administration of iNO. There was no interval improvement in median oxygen requirement (100% vs. 98% [86–100], *p* = 0.35) or oxygenation index (37.2 ± 9.0 vs. 41.6 ± 21.6, *p* = 0.53) after iNO administration (prior to initiation of milrinone).

Infants received an intravenous loading dose of milrinone (50 μg/kg) over 60 min followed by a maintenance infusion (0.33–0.99 μg/kg/min) for 24–72 h. Serial blood milrinone levels were collected after the bolus, following initiation of the maintenance infusion to determine steady state levels, and following discontinuation of the drug to determine clearance. Echocardiography was performed before and after (1, 12 h) milrinone initiation. The mean (SD) gestational age and weight at birth were 39.2 ± 1.3 weeks and 3481 ± 603 g. The median dose and duration of milrinone following the bolus were 0.33 μg/kg/min (range 0.33–0.99 μg/kg/min) and 24 h (range 24 to 42 h). The mean (SD) half-life, total body clearance, volume of distribution, and steady state concentration of milrinone were 4.1 ± 1.1 h, 0.11 ± 0.01 L/kg/hour, 0.56 ± 0.19 L/kg, and 290.9 ± 77.7 ng/ml.

The initiation of milrinone led to an improvement in PaO_2_ (*p* = 0.002) and a sustained reduction in FiO_2_ (*p* < 0.001), oxygenation index (*p* < 0.001), mean airway pressure (*p* = 0.03), and inhaled nitric oxide dose (*p* < 0.001). Although a transient reduction in systolic arterial pressure (*p* < 0.001) was seen following the bolus, there was overall improvement in base deficit (*p* = 0.01) and plasma lactate (*p* = 0.04) with a trend towards lower inotrope score. Serial echocardiography revealed lower pulmonary artery pressure, improved right and left ventricular output, and reduced bidirectional or right-left shunting (*p* < 0.05) after milrinone treatment. No infants were withdrawn from the study and there were no cases of intraventricular haemorrhage, electrolyte disturbances, abnormal liver or coagulation profiles, thrombocytopenia, need for ECMO or mortality.

### Clinical effects of milrinone in PPHN

In our centre (Rotunda Hospital, Dublin), infants with a clinical diagnosis of PPHN who fail to respond to iNO within 4 h of commencement undergo a comprehensive echocardiogram to rule out congenital heart disease, assess myocardial function and the degree of pulmonary hypertension (PH). Those infants are then commenced on milrinone in an attempt to augment iNO action and improve myocardial performance. We conducted a review of all infants undergoing this treatment over an 18-month period. The Rotunda Hospital Research Ethics Committee approved this study [28].

This was a retrospective review of infants ≥34 weeks gestation with PPHN who received milrinone. The primary end point was the effect of milrinone on RV and LV function parameters including right (RVO) and left (LVO) ventricular outputs, tissue Doppler velocities, RV and septal strain and strain rate, tricuspid annular plane systolic excursion (TAPSE) and LV myocardial performance index (MPI). Secondary endpoints examined included duration of iNO and oxygen support, change in blood pressure, and the use of cardio-respiratory support.

Seventeen infants with a mean (SD) gestation and birth weight of 39.8 (2.0) weeks and 3.45 (0.39) Kg respectively were included. All infants received iNO within 3 h of birth. The median duration of invasive ventilation for the cohort was 5 [5–8] days. The median hospital stay in the tertiary NICU was 12 [11–16] days. One infant died before discharge (35 ^5/7^ weeks gestation, polycystic kidney disease, prolonged anhydramnious and hypoplastic lungs). None of the infants required ECMO. The cohort underwent the first echocardiogram 15 [8–28] hours after iNO commencement. Milrinone was started at a median time of 1 [0.5–3] hour after the echocardiogram, 17 [9–34] hours after iNO treatment. The median [range] starting dose was 0.50 [0.3–0.66] μg/kg/min. Most infants remained on the median dose for the duration of the treatment with the exception of one infant requiring an escalation to 0.66 μg/kg/min and two up to 0.75 μg/kg/min. The median duration of treatment was 88 [65–118] hours.

Milrinone administration was associated with an increase in LVO (*p* = 0.04), RVO (*p* = 0.004), RV strain (*p* = 0.01) and strain rate (*p* = 0.002), and LV s` (*p* < 0.001) and a` (*p* = 0.02) tissue Doppler velocity waves. There was a significant reduction in iNO dose, oxygen requirement over the subsequent 72 h (all *p* < 0.05). In addition, the administration of milrinone was associated with a reduction in mean blood pressure (*p* = 0.04) peaking at 6 h post administration. This was associated with a significant increase in the use of vasoactive inotropes at 6 and 24 h. However, blood pressure began to increase after 12 h of milrinone administration with a peak at 72 h (systolic BP *p* = 0.02, mean BP *p* = 0.03, diastolic BP *p* = 0.02) in spite of a reduction in the use of vasoactive inotropes over the same time period.

### Rationale for milrinone use

PPHN is common and leads to a major burden of neonatal illness, carries a significant mortality, need for ECMO, and adverse neurodevelopmental outcome. There is poor appreciation of the physiologic determinants of PPHN and predictors of iNO response at presentation; up to 40% of infants do not respond to iNO treatment. The increasing cost of iNO may preclude its use in both the developed and developing countries. The use of milrinone in infants with PPHN as an adjunct to iNO is worth further exploration with preliminary evidence suggesting an improvement in both oxygenation and myocardial performance in this group of infants. It’s inotropic, lusitropic and pulmonary vasodilator properties make it an ideal agent to use in this setting and may improve response to therapy and reduce mortality associated with the disease.

A recent Cochrane review illustrated the lack of randomised controlled trials (RCTs) comparing the use of milrinone versus placebo or as an adjunct to iNO compared with iNO alone in the setting of PPHN and recommended limiting the use of milrinone in PPHN to the research setting [29]. It is important to systematically investigate the efficacy of milrinone in the setting of PPHN prior to widespread dissemination of this treatment.

### Objectives

#### Primary objective

We hypothesize that intravenous milrinone used in conjunction with iNO results in the reduction in the time on iNO therapy and the time spent on invasive ventilation in infants ≥34 weeks gestation and ≥ 2000 g with a clinical and echocardiography diagnosis of PPHN.

#### Secondary objective

The aim is to compare: the incidence of the use of other inotropes; critically low LV and RV function and output measured by echocardiography and a non-invasive cardiac output monitor (NICOM); the rate of adverse effects associated with milrinone including the incidence of hypotension; and the pre-discharge outcomes in the two groups. In this pilot study, we aim to assess the practicality of instituting the protocol and contributing to a sample size calculation for a definitive multicentre study.

### Trial design

**Fig. 1:**
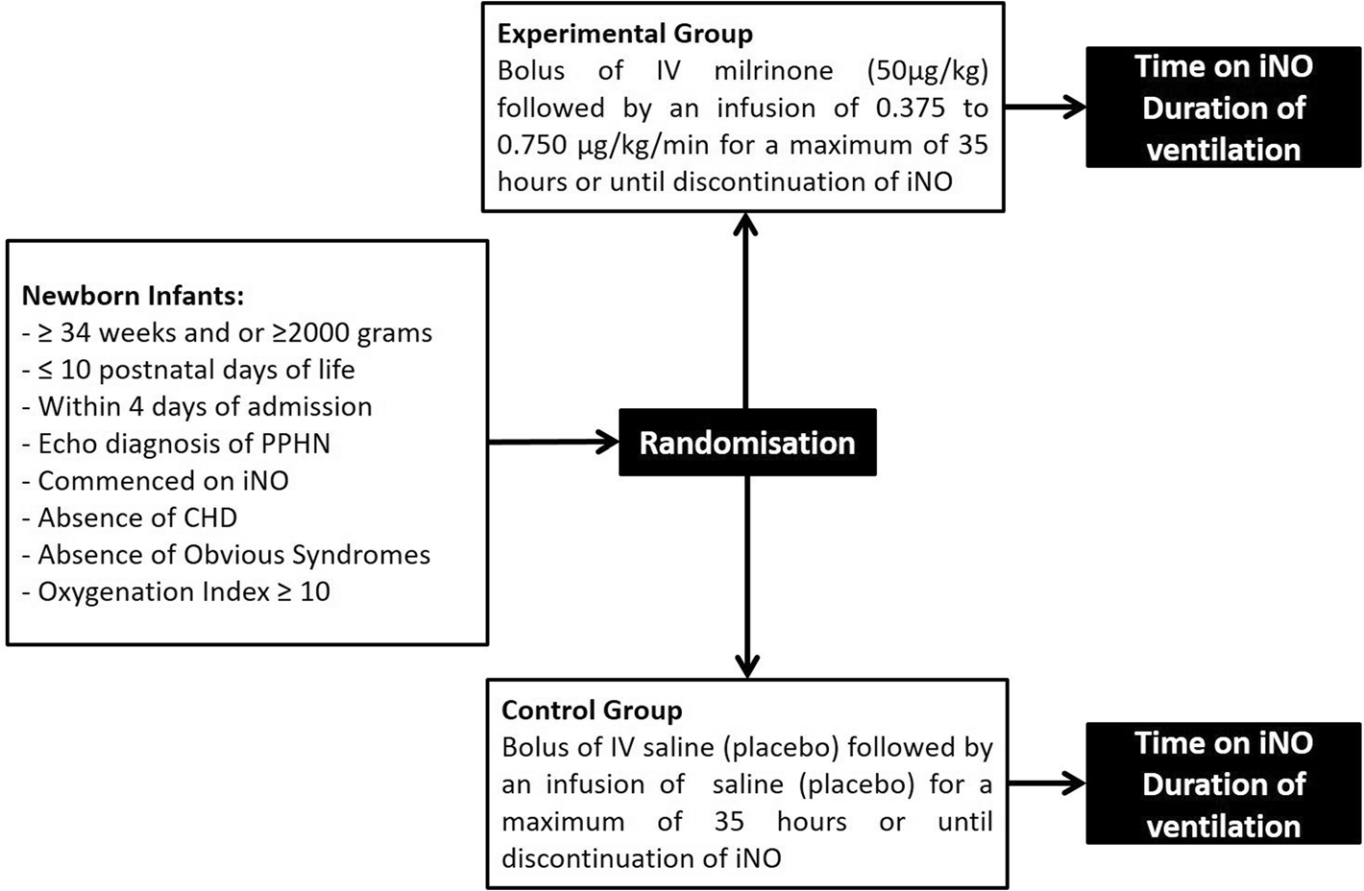
Trial Schematic

This is a multicentre, randomized, double-blind, two arm pilot study, with a balanced (1:1) allocation that will be carried out in the level III neonatal intensive care units in 2 centres in Ireland: The Rotunda Hospital Dublin and Cork University Maternity Hospital, and one centre in the Netherlands: Radboudumc Amalia Children’s Hospital, Nijmegen (Fig. 1).

### The relevance of the primary outcome

iNO use is expensive and currently requires invasive ventilation. Invasive ventilation can cause baro- and volutrauma, may lead to parenchymal lung damage and the evolution of pneumothoraces. Ventilation is often accompanied by the administration of sedative agents such as morphine and midazolam. In addition, prolonged ventilation will delay bonding with parents, delay feeding and suckling and will lead to prolonged hospitalisation. A reduction in the time spent invasively ventilated will (in addition to the other benefits outlined above) reduce hospital stay, promote earlier enteral nutrition, parental bonding, breast feeding and significantly reduce healthcare costs.

The purpose of this pilot study is to assess the feasibility of performing a pivotal trial and to obtain preliminary data to calculate a sample size for a definitive multi-centre trial of milrinone therapy in PPHN. We aim to recruit a total of 20 infants with PPHN (10 receiving milrinone and 10 receiving placebo) over a 4-year period. We then plan to seek additional funding in order to facilitate the running of the multicentre RCT in European and North American centres. In this pilot study we also aim to ascertain issues with recruiting infants and obtaining consent, compliance with the protocol, the rate (if any) of loss to follow up, and the general acceptability, feasibility and compliance of administering the intervention. This number will also allow us to calculate a standard deviation which will be used in a sample size calculation for the full-scale trial. In addition, we aim to refine the criteria for discontinuing therapy which are outlined below.

## Methods

### Participants interventions and outcomes

#### Setting

This feasibility study will be conducted in three Level III neonatal intensive care units (NICU). Two in Ireland: The Rotunda Hospital, Dublin, and Cork University Maternity Hospital; and one in the Netherlands: Radboudumc Amalia Children’s Hospital, Nijmegen.

#### Eligibility criteria

All infants with a gestation ≥34 week and a birth weight ≥ 2000 g admitted to the neonatal intensive care unit with a clinical diagnosis of PPHN and commenced on iNO will be deemed potentially eligible for this study. The process of iNO initiation in PPHN on clinical grounds and is standardised in the NICUs and is detailed in Additional file 1. The decision is made by the attending physician. In addition; the infants must satisfy the following criteria:

1. ≤ 10 postnatal days of life and within 4 days of admission
2. Echocardiography diagnosis of PPHN (see below)
3. Absence of significant congenital heart defect excluding a small atrial septal defect or ventricular septal defect (measuring less than 3 mm)
4. Oxygenation index of ≥10 measured obtained from an arterial blood sample and calculated using the following formula: OI = (Mean airway pressure × FiO_2_ × 100) ÷ (paO_2_ in kilopascals)

#### Exclusion Criteria

1. Lethal congenital anomalies or obvious syndrome
2. Bleeding diathesis (abnormal coagulation screen/platelet < 100,000/ mm^3^)
3. The presence of Intraventricular haemorrhage.
4. Diastolic Hypotension (defined as a diastolic blood pressure less than the 3rd centile for any given gestation) unresponsive to medical treatment (≥30 mL/kg fluid bolus and ≥ 2 inotropes)
5. Hypoxic-ischemic encephalopathy undergoing therapeutic hypothermia
6. Evidence of renal impairment (Creatinine >100 micromol/l)
7. Severe Hypovolaemia: Heart rate > 180, capillary refill > 5 s, urine output < 0.5 ml/kg/hour, in addition to diastolic hypotension mentioned above.

#### Intervention

Infants in the intervention arm will receive an intravenous loading dose of milrinone lactate injection (10 mg/10 mL) at a dose of 50 μg/kg administered over 60 min followed by a maintenance infusion, beginning at 0.375 μg/kg/min to a maximum 0.750 μg/kg/min. The duration of therapy is until discontinuation of iNO or a maximum of 35 h in adherence with the summary of product characteristics (SmPC) recommendation. A 10 mL/kg bolus of normal saline will be administered with the milrinone bolus over the same 1 h period.

A loading dose was chosen to ensure the rapid attainment of therapeutic blood concentrations. The slow rate of administration was chosen to minimize the risk of infusion-related adverse effects, such as hypotension. This dosing regimen was established following the recent pharmacokinetic study performed by our group and described above [27]. Dose increase will be performed in response to the need for oxygen (FiO_2_) to maintain adequate pre-ductal saturations (95% or greater). Dose incrementation will be carried out every 2 h following the bolus if the fall in FiO_2_ needed to maintain an adequate saturation is less than 20%. Dose escalation will be carried out as described below (Section “Product and placebo preparation and infusion regimen”). For example, if 2 h after administration, the FiO_2_ of an infant falls from 60 to 45% (< 20%) to maintain a saturation of 95% or greater, the rate of infusion will be increased based on the table below. This escalation will continue every 2 h until a reduction of FiO_2_ of 20% or greater is achieved down to 40% FiO_2_. The maximum dose will be 0.750 μg/ kg/min.

#### Control

Infants in the control arm will receive an intravenous loading dose of placebo (normal saline) at a rate equivalent to the infusion rate of the milrinone bolus, administered over 60 min. A bolus of normal saline of 10 ml/kg will accompany the placebo infusion, as per the intervention arm. Following the loading protocol, a saline infusion running at a rate similar to the milrinone infusion will be commenced for a maximum period of 35 h or until discontinuation of iNO if it occurs sooner. The saline infusion will be titrated up in increments similar to the milrinone infusion.

#### Product and placebo preparation and infusion regimen

The proposed placebo is an authorised product in the EU- see PA below.We will prepare the product in line with the instructions in the SPC. The outline below will be included in the protocol as suggested. The product will be diluted with solutions specified in section 6.6 of the SPC. A milrinone infusion solution containing 200 micrograms/ml will be prepared by diluting 10 ml of Primacor injection with 40 ml of 0.9% sodium chloride (Baxter Sodium Chloride 0.9% *w*/*v* Intravenous Infusion BP 100 ml- PA 0167/008/015). The syringe will be inverted at least 10 times to ensure adequate mixing. Each infusion syringe will be freshly prepared before use. The prepared infusion will be visually inspected for particulate matter or discolouration. The diluted solution will be used within 1 h of preparation.

The placebo infusion will consist of 50 ml of the diluent (Baxter Sodium Chloride 0.9% w/v Intravenous Infusion BP 100 ml- PA 0167/008/015). Each placebo infusion syringe will be freshly prepared before use. The prepared placebo infusion will be visually inspected for particulate matter or discolouration. Administration rates will be guided by the SPC:

**Table:** 

**Primacor Injection Dose (μg/kg/min)**	**Infusion Delivery Rate (ml/kg/hr)**
0.375	0.11
0.400	0.12
0.500	0.15
0.600	0.18
0.700	0.21
0.750	0.22

#### Use of additional inotropic and concomitant therapy

The most common co-morbidity accompanying pulmonary hypertension in term infants is hypotension. The approach to managing systemic hypotension will be as follows:

- Systolic hypotension (defined as a systolic BP < the 3^rd^ centile for any given gestation) will be treated with a bolus of saline (10 ml/kg) followed by adrenaline at a dose of 0.05 μg/kg/min up to 2 μg/kg/min.
- Diastolic hypotension (defined as a diastolic BP < the 3^rd^ centile for any given gestation) will be treated with a bolus of saline (10 ml/kg) followed by dopamine at a dose of 5 μg/kg/min up to a maximum of 20 μg/kg/min.

Infants failing to respond to the above interventions will be withdrawn from the study and un-blinded (see below). Dobutamine will not be used during the study period due the risk of hypotension when used concomitantly with milrinone. Adrenaline/noradrenaline will be used as second line agents. Steroids will be used for severe hypotension/shock at a dose of 1 mg/kg 6 hourly IV.

If required, infants will be sedated with fentanyl (rather than morphine) to avoid the risk of hypotension. The starting dose of the fentanyl infusion is 1.0 μg/kg/min following a bolus of 1.0 μg/kg. Management of all other aspects of pulmonary hypertension will be at the discretion of the attending neonatologist based on unit policies. Other pulmonary vasodilator agents such as sildenafil, prostacyclin and/or bosentan will not be used during the study period.

Other considerations:

- Aminophylline/Caffeine: aminophylline/caffeine may compete with milrinone for phosphodiesterase inhibition and therefore may attenuate the clinical effect. However, aminophylline/caffeine administration is not used in infants with PPHN and therefore the risk of co-administration is low. None-the-less, we will exclude patients with concomitant administration from the study.
- Frusemide or bumetanide will not be administered in intravenous lines containing milrinone lactate in order to avoid precipitation.
- Milrinone will not be diluted with sodium bicarbonate.
- As outlined below in the monitoring section (Monitoring, outcome measures and participant timeline): hypokalaemia will be monitored and corrected in advance of/and during milrinone use as diuresis secondary to improved cardiac output may ensue following milrinone administration.
- Calcium channel blockers (CCB) are not used in neonates and therefore the potential interaction between milrinone and CCBs is not a concern in this study.

#### Exit criteria

The following criteria will mandate the discontinuation of the intervention in both arms and a return to standard of care, as these are considered side effects of the medication if they occur within 1 to 3 days of administration of the medication: profound hypotension requiring > 30 mL/kg fluid bolus and inotropic support of ≥20 mcg/kg/min of intravenous dopamine or dobutamine, thrombocytopenia of ≤100,000/mm3, and arrhythmia requiring treatment.

#### Randomisation and allocation

A computer-generated central randomisation scheme will be used to assign the infants to the two arms in a 1:1 ratio. Each infant will be assigned a unique 3 digit identification (ID) number. The study pharmacist in each centre will receive a binder containing the sequence of treatment group assignments for the cohort from a statistician who will not otherwise be involved in the study. At each study center, access to the binder will be restricted to selected pharmacy personnel, and will be kept locked in a secure locker in the pharmacy department.

#### Concealment and blinding

Both formulations are clear and colourless. The ampoules used will be identical and will have a study identification number included in the label on the ampoule. This number will be known to the study pharmacist. Once a patient is recruited and randomised to either Milrinone or Placebo, the trial pharmacist or physician not involved in recruitment, allocation, and data collection will prepare the trial drug or placebo and issue the syringe for infusion to the trial investigator team for administration. An on call arrangement will be devised for out of hours preparation. The milrinone preparation is colourless and odorless and will be indistinguishable from the saline preparation used for the placebo arm. The designated pharmacist in each study centre will be aware of the treatment allocation in order to facilitate correct assignment and drug/placebo preparation. The trial participants and their families, the care providers, the data collectors, the echocardiographers, the primary outcome assessors, and the data analysts will all be blinded to the allocation.

#### Monitoring, outcome measures and participant timeline

The primary outcome of this study is the time on iNO in hours and the time on invasive ventilation. A standardised protocol for iNO weaning is detailed in Additional file 2.

We will collect the following demographic characteristics and relevant short-term secondary outcomes: duration of hospital stay; time to extubation; duration of oxygen therapy, and survival.

**Details of cardiorespiratory stability** (blood pressure, heart rate, oxygen saturation), ventilation support (FiO_2_, mean airway pressure (MAP), oxygenation index [MAP × F FiO_2_/PaO_2_]), efficacy of oxygenation [PaO_2_], plasma lactate, pH, PCO_2_, HCO_3_^−^, Base excess; and relevant co-treatments (i.e., sedation, analgesics, muscle relaxants, inotropes, fluid administration) at the following time points:

1. Prior to treatment
2. Following the loading dose
3. 12 h after initiation of therapy
4. 24 h after initiation of therapy
5. Two hours after discontinuation of therapy

**Monitoring of blood parameters** will be carried out as follows during the study period and 24 h after drug discontinuation:

- Full blood count (including platelets): daily
- Electrolytes, Urea and Creatinine: daily
- Liver function tests (ALT, AST, GGT): daily
- Blood gas analysis: 12 hourly

**Echocardiography** will be performed according to the following schedule: a timing window of ±3 h will be applied. Scans performed outside the ±3 h window will constitute a protocol deviation:

1. Prior to the Commencement of Study Drug: Diagnose PPHN and rule out CHD
2. 12 h following the administration of Study Drug
3. 24 h following the administration of the study drug
4. 8 h following the discontinuation of the study drug

**Non-invasive Cardiac output Monitoring (NICOM)** will be commenced prior to the commencement of the study drug and continued until the last echocardiogram, 8 h after the discontinuation of the study medication.

**Cranial ultrasound** will performed prior to treatment commencement and after the discontinuation of the infusion. Intraventricular haemorrhage will be defined and graded with the Papile classification [30]. Echocardiography will be carried out at baseline as detailed above and at 12 h, 24 h, 2 h after discontinuation of therapy, and before discharge.

#### Echocardiography assessment of PPHN

All infants will undergo a comprehensive echocardiography assessment at baseline prior to enrollment to rule out congenital heart disease, quantify the magnitude of pulmonary hypertension and assess left and right ventricular output and function. The functional protocol for the echocardiography assessment is adapted from the recent American Society of Echocardiography Guideline on neonatal echocardiography [31, 32]. All infants will be assessed once established on iNO and are in a quiet state using the Vivid E9 or the Vivid S6 (GE Medical, Milwaukee, WI, USA) and a 10 Mhz transducer. A diagnosis of PPHN will be made if any of the following is identified in the absence of cyanotic congenital heart disease:

- A tricuspid regurgitant jet with a pressure gradient ≥^2^/_3_ systemic systolic blood pressure
- Intra-ventricular septum flattening or bowing into the left ventricular cavity
- Patent ductus arteriosus bidirectional shunting or predominant right to left shunting
- A pulmonary artery acceleration time < 40 milliseconds

We will collect the following parameters at the same time points outlined in section “Monitoring, outcome measures and participant timeline”:

- *Assessment of pulmonary Hypertension*: PDA Shunt Direction; Pulmonary artery acceleration time (PAAT); Right ventricular ejection time (RVET); PAAT:RVET ratio; Tricuspid regurgitant jet pressure gradient; Interventricular septum motion in systole.
- *Assessment of RV performance*: Right ventricular tissue Doppler velocities, strain and strain rate; Right ventricular output (RVO); Right ventricular TAPSE (Tricuspid Annular Peak Systolic Velocity); Right ventricular dimensions; Right ventricular fractional area shortening.
- *Assessment of LV performance*: Shortening fraction; Ejection fraction measured by Simpson’s biplane method; Tissue Doppler velocities; Left ventricular strain and strain rate; Left ventricular output (LVO); Left ventricular twist, and twist/untwist rate.
- *Assessment of Pulmonary venous return*; Left atrial to aortic root ratio; Pulmonary vein peak systolic and diastolic velocity; Mitral valve E and A velocities, E:A ratio, and mitral value VTI, IVRT; The presence of PFO/ASD and the velocity of the shunt.
- *Assessment of PDA parameters*: PDA size in 2D measured at the pulmonary end, mid ductal and aortic end; the direction and peak velocity of flow across the shunt; Pressure gradient across the shunt.
- *Markers of systemic hypoperfusion*: Abdominal aortic peak systolic velocity and diastolic flow direction and velocity; Celiac artery peak systolic velocity and diastolic flow direction and velocity; Middle cerebral artery peak systolic velocity and diastolic flow direction and velocity.

Reliability analysis was recently conducted and ascertained normative data for all the above-mentioned echocardiography parameters in 50 healthy term infants. Acceptable reliability was demonstrated in measuring all the functional parameters. In addition, the reference ranges and the effect of physiological postnatal transition is described [33].

Slow closure of the patent ductus arteriosus is a theoretical risk in association with the use of milrinone due to the drug’s vasodilatory properties. However, in our observational study of 17 infants (section 1.2.3), none had a patent ductus arteriosus by the time of discharge despite the relatively longer duration of milrinone use in the observational study (median of 88 h). We will assess the presence of patent ductus arteriosus before discharge on all infants enrolled to further study this association.

#### Non-invasive cardiac output assessment (NICOM)

Non-invasive cardiac output monitoring has gained interest in the assessment of neonatal haemodynamics [34, 35]. Trans-thoracic bioreactance is a new technique of non-invasive continuous cardiac output monitoring. It is based on an analysis of relative phase shifts of oscillating currents that occur when an injected current traverses the thoracic cavity. The system’s signal processing unit determines the relative phase shift (Φ) between the input and output signals. Stroke volume (SV) can be estimated by: SV = C x VET x dΦ/dtmax, where C is a constant of proportionality, VET is ventricular ejection time (determined from the NICOM and ECG signals) and dΦ/dtmax is the peak rate of change of Φ.

The value of C has been optimized in prior studies and accounts for patient age, gender and body size. SV and left ventricular (LVO) measurement are provided in 60 s intervals [36]. In the adult population, NICOM has acceptable accuracy and precision for cardiac output monitoring in patients with hemodynamic instability when compared to thermodilution, aortic artery catheterisation, and cardiopulmonary bypass pumps [36–38]. Recently, NICOM was compared to echocardiography measures of LVO in a cohort of near term and term neonates with acceptable reliability and precision [35]. In addition, NICOM has the ability to identify different haemodynamic patterns and response to milrinone administration in preterm infants following PDA ligation [35]. These properties suggest that NICOM may be an ideal device for the assessment of the haemodynamic profile of infants with PPHN.

Continuous LVO measurement using bioreactance is facilitated by the NICOM system. The measurement will take place during the entire study period. Bioreactance is the analysis of the variation in the frequency spectra of a delivered oscillating current when it traverses the thoracic cavity. This is obtained by placing four emitting and receiving electrodes in a manner that “boxes” the heart. Each electrode sensor strip consists of two contact points. Upper thoracic electrode strips will be placed over the mid-clavicles and upper back bilaterally. The lower electrode sensors are placed between the 6th and 7th intercostal spaces at the mid-axillary line. The electrode set will be replaced after 48 h of measurement to ensure adequate signal acquisition. NICOM measurement of SV and LVO will be blinded to the echocardiography and the clinical team by covering the screen displaying the values. The echocardiographic measurement of SV and LVO will be paired with the corresponding NICOM readings following study completion. The NICOM system stores each reading of SV and LVO. SVR will be measured by the NICOM system on an hourly basis as the mean blood pressure is entered into the machine.

### Data management and statistical analysis

#### Sample size

This study is conducted to determine the feasibility of patient recruitment, instituting the study protocol, randomising and blinding allocation, collecting outcome data and contribute to determine the sample size necessary for a definitive multicentre trial. A sample of 10 infants per arm (a total of 20 infants) will be recruited over a 4-year period.

#### Recruitment strategies

The recent incidence of pulmonary hypertension in the three study sites is 1–2 per 1000 live births. The total annual birth rate in the 3 centres is around 24,000 giving 24–48 potential eligible infants. The rate of moderate to severe PH in this cohort (fulfilling the eligibility criteria) will be around 50% (12–24 infants per annum). We anticipate a 50% to 75% enrolment rate per year to account for consent refusal, competition with other studies and unavailability of staff to recruit patients. Therefore, we anticipate a recruitment period of 4 years. All centres are experienced in the conduct of clinical trials. They also have the expertise to carry out the assessment of the primary and secondary outcomes and to perform the echocardiography and NICOM assessments. We do not anticipate any variability in administering the intervention between sites over time.

#### Data management

Clinical and demographic data, as well as outcome data will be collected by the research fellows at each site who will be blinded to treatment allocation. Pilot testing of the data collection sheet will be performed prior to study commencement. No patient identifiers will be used and each patient will be assigned a unique ID number based on the sequence in recruitment. The data sheets will be stored in a designated secure locker with access granted only to the research fellows and principal investigators. Echocardiography data will be stored on secure hospital servers. Off-line analysis of the echocardiography data will be performed and image analysis techniques will be standardised. The investigators analysing the study will be blinded to the allocation.

#### Statistical methods

The HRB IP CTN clinical trial statistician will conduct all the statistical analyses. The primary outcome and most of the secondary outcomes are continuous variables. Continuous variables will be tested for normality by comparing the mean and median, a histogram representation of data, and the Shapiro-Wilk test for normality and will be presented as means (standard deviation) or median [inter-quartile range] as appropriate. Dichotomous variables will be presented as proportions and summarised in contingency tables. The *primary analysis of the primary outcome* will be performed using an independent t-test (or a Wilcoxon Rank Sum test as appropriate) to compare the difference in time on iNO and invasive ventilation between the two groups. A chi squared test (or Fisher’s exact test) will be used for the *primary analysis of the dichotomous secondary* outcomes. For the continuous secondary outcomes, a t-test will be used to compare normally distributed data, and Wilcoxon Rank Sum test will be used for skewed data. We will accept a *p* value of < 0.05 as significant. We will use SPSS (version 22) to perform the statistical analysis.

All enrolled infants will be analysed on an intention-to-treat basis, including infants that have not continued treatment for any reason. Analysis for the feasibility study will only be conducted once the recruitment of all patients is completed. No interim analysis of treatment effect will be conducted. We anticipate that recruitment will take 4 years to complete with an additional 6 months required for the measurement of the echocardiography data and statistical analysis.

### Data monitoring and managing adverse effects

The study will be registered online at EUDRACT. The day-to-day management of the study will be coordinated by a designated research fellow at each study site. The research team in each site will consist of the following personnel: Principal investigator with echocardiographic skills designated study pharmacist for drug preparation; research fellow for consent, infant enrolment and data collection.

A Trial Management Committee (TMC) will be set up to include the lead Principal Investigator, HRB IP CTN statistician, Quality and Regulatory Manager and Trial coordinator. This committee will be responsible for: integration of the study protocol into the clinical setting; training of the various study personnel; adherence to study protocol; maintenance of concealment and adequate randomisation. In addition, the TMC will oversee data collection and ensure completeness, validity, and track recruitment rates in the various sites in collaboration with the Trial Sponsor (The Royal College of Surgeons in Ireland) Data checks will include assessment of the presence of missing or invalid data.

The conduct of this study will conform to the ICH Harmonized Tripartite Guideline for Good Clinical Practice. An independent Data Monitoring Committee (DMC) will be convened and consist of 2 external neonatologists and a statistician. This will be independent from the sponsor (The Royal College of Surgeons in Ireland) and from competing interests. The role of this committee will consist of monitoring the emergence of adverse effects and any unexpected hazards.

## Ethics, dissemination

Ethical approval will be sought from the local research ethics committees at all centres. HPRA approval for the use of milrinone in PPHN will be sought prior to commencement of the study and any amendments to the protocol will be submitted to both the HPRA and the research ethics committee. The Dutch site will seek local regulatory body approval in the Netherlands.

The local PI will initially approach the physician responsible for the care of the infant for permission to approach the parents with a view to informing them of the pilot study. All parents will be provided with a clear explanation of the objectives, procedures, risks and benefits of the study in the Parent Information Leaflet. The investigator must provide time and opportunity for them to ask questions and ascertain details of the study. Further information will be given verbally and all questions will be answered. Parents will be given 6 h to consider enrolment into the study. Once all discussions are completed they will be invited to sign the consent form.

Informed consent will be obtained before any study assessments/procedures are performed and before any data collection occurs. All personal study participant data collected and processed for the purposes of this study will be managed by the investigators and their staff with adequate precautions to ensure the confidentiality of those data and in accordance with applicable national and local laws and regulations on personal data protection. The ethics committees approving this research will be granted direct access to the study participants’ original medical records for verification of clinical trial procedures and/or data, without violating the confidentiality of the participants, to the extent permitted by the law and regulations.

No patient identifiers will be used during data collection or analysis of the study. No individual patient data will be presented. All data will be presented summarized as means, medians, or proportions as appropriate. Patients will be allocated a unique number based on site and enrolment number. Parents are free to withdraw their infants from the research study at any stage. Data will be stored in an encrypted secure computer in a locked office in the department of Paediatrics.

The results of the study will be shared with all participant families. In addition, they will be presented at all the participating hospitals’ grand rounds. Data will be presented at the Annual Pediatric Academic Society Meeting for mass dissemination of the results. Submission for publication to a peer reviewed journal of a high impact factor will also be sought.

## Conclusion

### Significant savings for the health service

A positive outcome in this pilot study will pave the way for a definitive trial of the use of milrinone for the treatment of PPHN. Improving the response rate to iNO and reducing the time on iNO and mechanical ventilation will have substantial short and long-term benefits for the healthcare service. Reduced length of stay will significantly reduce hospital costs in the neonatal intensive care unit. In addition, iNO administration is expensive (up to €50 per hour). Milrinone is relatively cheap costing about €30 per patient. There is potential for substantial savings if the use of milrinone is successful in reducing time on iNO. The increasing cost of iNO may soon preclude its use in both developed and developing countries so this study is urgently needed.

### Reducing burdens on families

Improving the response rate to iNO, reducing the time on iNO and mechanical ventilation will have substantial short and long-term benefits for the infants and their families. Reducing hospital stay, improving the response rate and reducing the chance of undergoing ECMO will improve the quality of life of the infant, facilitate earlier nutrition and discharge, and potentially improve neurodevelopmental outcome.

### A greater understanding of the pathophysiology of PPHN

PPHN is a common occurrence in neonates and leads to a major burden of illness, carries a significant mortality, a need for ECMO, and adverse neurodevelopmental outcome. There is poor appreciation of the physiologic determinants of PPHN and predictors of iNO response at presentation with up to 40% of infants not responding to iNO treatment. The echo and NICOM measurements performed in this study will provide evidence both on the pathophysiology of PPHN but also on the efficacy of milrinone therapy.

### Establishing Ireland as a recognised leader in cardiovascular support

This pilot study will be the first randomised controlled trial to evaluate milrinone use in term infants with pulmonary hypertension. This study, along with another HRB IP CTN trial - the HIP Trial which evaluates blood pressure support in preterm infants, again the first of its kind, will establish Ireland’s neonatal community as world leaders in the area of newborn cardiovascular support.

## Additional files

Additional file 1: Commencement of iNO in infants with PPHN. (DOCX 39 kb)

Additional file 2: Weaning iNO. (DOCX 43 kb)
