# Supplementary material for: The use of milrinone in neonates with persistent pulmonary hypertension of the newborn - a randomised controlled trial pilot study (MINT 1): study protocol and review of literature
Source: Matern Health Neonatol Perinatol. 2018 Dec 3;4:24. doi: 10.1186/s40748-018-0093-1 (PMC6276183; doi:10.1186/s40748-018-0093-1)
Supplement: Supplementary file 1 — Commencement of iNO in infants with PPHN. (DOCX 39 kb) [file 40748_2018_93_MOESM1_ESM.docx]

**Additional file 1: Commencement of iNO in infants with PPHN**

**Before starting iNO** (Optimizing patient condition): All possible attempts should be made to address the following before iNO is started in any patient –

Mean airway pressure is adjusted to provide adequate lung inflation and must be confirmed by chest X-ray. In many but not all situations, this might mean using high frequency ventilation (HFOV) particularly if patient also has high PaCO2 and parenchymal lung disease. HFO & iNO should not be started simultaneously. HFO (if deemed appropriate) iNO and settings adjusted to correct hypercapnia. Time should be given to adjust/assess its impact before iNO is started.

**Correct acidosis and hypercapnia**. Ensure PaCO2 and pH are in clinically acceptable range before starting iNO. In most situations the aim should be for PaCO2 to be 4.6 – 6 kPa (35 – 45 mmHg) and for pH to be between 7.30-7.40 (not less than 7.25). Correction of metabolic acidosis can be through use sodium bicarbonate or by fluid resuscitation if poor perfusion is simultaneously present.

**Optimize sedation** with or without muscle relaxant to ensure patient is as comfortable as possible and agitation is not a significant contributor to high PVR. **Establishment of adequate sedation is vital prior to muscle relaxation.**

**Correct hypo-perfusion** if present by appropriate fluid resuscitation with saline to a maximum of 30 ml/kg.

**Starting iNO**:

- Consider iNO if the oxygenation index (OI) is ≥ 10
- Ensure airway is cleared with suctioning prior to starting iNO
- Always start at 20 parts per million in a term infant (ppm)
- Document onto patient flow sheet start time of iNO
- **DO NOT** make any ventilatory/FiO2 changes or disconnect infant from ventilator for at least 30 minutes (or less provided a positive response is established) after iNO has been started to be able to establish patient’s responsiveness to treatment.

**Assessing response**: The iNO responsiveness **must** be reassessed within 30-60 minutes following initiation. It is preferable to obtain another ABG and establish response using PaO2 but in cases where arterial access is not available post-ductal or SpO2 may be used.

| **Positive response** | Rise in post-ductal PaO2 ≥ 2.5 KPa or ≥ SpO2 by 10% (or able to drop FiO2 by at least 0.2) |
| --- | --- |
| **Partial response** | Rise in post-ductal PaO2 1 – 2.5 KPa or SpO2 between 5- 10% (or able to drop FiO2 by 0.1 – 0.2) |
| **Negative response** | Either **no change** or rise in postductal PaO2 SpO2 by < 5% (or able to drop FiO2 by < 0.1) |

Follow the schematic below for further iNO treatment:

**Negative Response:**

- PaO_2_ rise < 1.0 KPa
- FiO_2_ requirement do not reduce

Allow 1 hour to assess response

Stop iNO.

Document response

**Partial Response:**

- Post ductal PaO_2_ rise 1.0 - 2.5 KPa
- FiO_2_ requirement fall by 10- 20 %
- FiO_2_ does not wean to 40%

Allow 1 hour to assess response

Maintain Sats 92 to 97% .Once optimal FiO_2_ is established iNO for 12 hours

**Prior to Staring iNO**

- Optimise lung Inflation
- Initiate HFO if deemed appropriate
- HFO and iNO should not be started simultaneously
- Correct Acidosis (pH 7.30 – 7.40)
- Correct hypercapnea (CO_2_ 4.6 – 6.0 KPa)
- Optimize sedation
- Correct Hypoperfusion
- Obtain post ductal ABG

**Initiation of iNO**

- Consider iNO if OI ≥ 15 in spite of above measures
- Start iNO if OI > 20
- Start iNO at 20 ppm for term infants
- NO ventilator changes
- NO FiO_2_ changes
- Monitor response.

**Positive Response:**

- Post ductal PaO_2_ rise > 2.5 KPa
- FiO_2_ requirement fall by 20%
- FiO_2_ Weans to 40%

Allow 1 hour to assess response

Maintain Sats 92 to 97% for term

Maintain sats 90 to 95% for preterm

Keep iNO for 12 hours once FiO2 requirements = or < 40%
