# Supplementary material for: The use of milrinone in neonates with persistent pulmonary hypertension of the newborn - a randomised controlled trial pilot study (MINT 1): study protocol and review of literature
Source: Matern Health Neonatol Perinatol. 2018 Dec 3;4:24. doi: 10.1186/s40748-018-0093-1 (PMC6276183; doi:10.1186/s40748-018-0093-1)
Supplement: Supplementary file 2 — Weaning iNO. (DOCX 43 kb) [file 40748_2018_93_MOESM2_ESM.docx]

**Additional file 2: Weaning iNO**

**Weaning iNO**: For infants who have had a positive response to iNO therapy, FiO_2_ should be weaned to a minimal tolerated concentration. After a stable period of 4 hours, the patient should be assessed for suitability for weaning iNO.

- Weaning of iNO should commence when FiO2 < 0.4.
- Wean iNO by 5 ppm q1h until down to 5 ppm followed by one of the following:
  - If total duration of iNO > 24 hours: leave on 5 ppm for 4 hours and then wean by 1 ppm q1h.
  - If total duration of iNO therapy < 24 hours: wean by 1 ppm q1H till off.

**Discontinue weaning if** at any point one or more of the following occur: FiO2 rise by > 0.2, return of pre-ductal saturation higher than post-ductal by > 10%.

If weaning had to be discontinued then increase iNO by one step at a time till infant returns back to pre-deterioration status and then leave for at least 12 hours before recommencing weaning. This time weaning strategy should be slower than earlier attempt and should be decided on an individual basis by the attending team.

Be aware that some infants may develop a transient hypoxemia once iNO therapy is discontinued. This is due to suppression of endogenous NO by exogenous therapy. The hypoxemia is usually moderate (needing increase in FiO_2_ by 0.2) and short lasting (up to an hour). **This should not be a reason to restart iNO therapy** but should be treated by increasing FiO_2_

**iNO weaning algorythm**

**Weaning iNO**

Infants with a positive or partial response to iNO for 4 hours are suitable for weaning

Commence NO wean when FiO_2_ < 0.4

**Increase iNO by 1 ppm until infant returns to pre-deterioration status**

**Discontinue weaning iNO if:**

- FiO_2_ increase by 0.20
- Pre / post ductal SpO_2_ difference > 10)

**Stop iNO and document duration of therapy**

**@5ppm: If duration of iNO is greater than 24 hours leave iNO on 5ppm for 4 hours THEN wean by 1ppm every 1 hour**

**@5ppm: If duration of iNO is less than 24 hours wean by 1ppm every 4 hours**

**Wean iNO by 5 ppm every 1 hour to 5 ppm**
